# Anti-TNF-α Activity of *Portulaca oleracea* in Vascular Endothelial Cells

**DOI:** 10.3390/ijms13055628

**Published:** 2012-05-10

**Authors:** An Sook Lee, Jin Sook Kim, Yun Jung Lee, Dae Gill Kang, Ho Sub Lee

**Affiliations:** 1 College of Oriental Medicine and Professional Graduate School of Oriental Medicine, Wonkwang University, Shinyong-dong, Iksan, Jeonbuk, 570-749, Korea; E-Mails: mama0099@nate.com (A.S.L.); shrons@wku.ac.kr (Y.J.L.); 2 Korea Institute of Oriental Medicine, Jeonmin-dong, Yusung-gu, Daejeon, 305-811, Republic of Korea; E-Mail: jskim@kiom.re.kr; 3 Hanbang Body-fluid Research Center, Wonkwang University, Shinyong-dong, Iksan, Jeonbuk, 570-749, Korea

**Keywords:** *Portulaca oleracea*, inflammation, NF-κB, reactive oxygen species (ROS), atherosclerosis

## Abstract

Vascular inflammation plays a key role in the pathogenesis and progression of atherosclerosis, a main complication of diabetes. The present study investigated whether an aqueous extract of *Portulaca oleracea* (AP) prevents the TNF-α-induced vascular inflammatory process in the human umbilical vein endothelial cell (HUVEC). The stimulation of TNF-α induced overexpression of adhesion molecules affects vascular cell adhesion molecule (VCAM)-1, intercellular adhesion molecule (ICAM)-1 and E-selectin for example. However, AP significantly suppressed TNF-α-induced over-expression of these adhesion molecules in a dose-dependent manner. In addition, pretreatment with AP dose-dependently reduced an increase of the adhesion of HL-60 cells to TNF-α-induced HUVEC. Furthermore, we observed that stimulation of TNF-α significantly increased intracellular reactive oxygen species (ROS) production. However, pretreatment with AP markedly blocked TNF-α-induced ROS production in a dose-dependent manner. The western blot and immunofluorescence analysis showed that AP inhibited the translocation of p65 NF-κB to the nucleus. In addition, AP suppressed the TNF-α-induced degradation of IκB-α and attenuated the TNF-α-induced NF-κB binding. AP also effectively reduced TNF-α-induced mRNA expressions of monocyte chemoattractant protein (MCP)-1 and interleukin (IL)-8 in a dose-dependent manner. Taken together, AP prevents the vascular inflammatory process through the inhibition of intracellular ROS production and NF-κB activation as well as the reduction of adhesion molecule expression in TNF-α-induced HUVEC. These results suggested that AP might have a potential therapeutic effect by inhibiting the vascular inflammation process in vascular diseases such as atherosclerosis.

## 1. Introduction

Recent investigations of atherosclerosis have focused on vascular inflammation, providing novel insight into mechanisms of disease. Much evidence has demonstrated that inflammatory events contribute at each stage in the development of clinically significant atherosclerosis [1,2]. The recruitment of circulating monocytes/leukocytes to inflamed sites in the arterial wall is one of the earliest detectable events in atherogenesis. Endothelial cells recruit leukocytes by selectively expressing major adhesion molecules on the surface, for example, vascular cell adhesion molecules (VCAM-1), intercellular adhesion molecules (ICAM-1), and endothelial cell selectin (E-selectin) [3]. The pro-inflammatory cytokine, tumor necrosis factor (TNF)-α, is a prototype “activation agonist” that modulates leukocyte adhesion and transmigration in vascular inflammatory diseases including atherosclerosis [4]. Previous studies have indicated that cytokines such as TNF-α change the shape and motility of endothelial cells, which could contribute to vascular leakage at the site of inflammation and that such cytokines stimulate an increase in the expression of cell adhesion molecules [5,6]. An increased expression of adhesion molecules by endothelial cells in human atherosclerotic lesions may lead to further recruitment of leukocytes to atherosclerotic sites.

TNF-α also activates signaling cascades that regulate the activation and translocation of redox-sensitive transcription factor NF-κB, an obligatory mediator of the inflammatory response that causes transcriptional activation of genes encoding adhesion molecules [7]. Previous studies have shown that NF-κB activation is required for the up-regulation of adhesion molecules such as ICAM-1 and VCAM-1, which are responsible for monocyte adhesion and increased vascular inflammation [8,9].

Increased oxidative stress in vascular cells is implicated in the pathogenesis of atherosclerosis. Reactive oxygen species (ROS) are important mediators in cellular injury, specifically, as a factor in endothelial cell damage. A potent factor in causing intracellular ROS formation in endothelial cells is TNF-α, a pleiotropic inflammatory cytokine [10]. In addition, ROS may serve as a common intracellular messenger for various redox-sensitive transcription pathways that induce adhesion molecule expression in vascular endothelial cells [2,11]. Recent studies showed that substances with antioxidant activity could scavenge intracellular ROS and inhibit endothelial adhesiveness to monocytes by reducing the expression of various adhesion molecules [12,13].

*Portulaca oleracea* L. (*Portulacaceae*) is an edible plant and has been used as a folk medicine in many countries, acting as a diuretic, febrifuge, antiseptic, antispasmodic and vermifuge [14,15]. It has been shown to display pharmacological roles, including antibacterial [16], analgesic [17], skeletal muscle-relaxant [18] and wound-healing [19] activities. Many studies have also shown that the major bioactive components of *Portulaca oleracea* are flavonoids, coumarins, monoterpene glycoside and alkaloids [20,21]. Some research results indicated that *Portulaca oleracea* could also be used to reduce the incidence of cardiovascular diseases [22]. However, there are no reports on the efficacy of an aqueous extract of *Portulaca oleracea* (AP) on either the expression of the cell adhesion molecules or the adhesion monocytes to endothelial cells through the NF-κB signaling pathway. Therefore we investigated the anti-vascular inflammatory effect and molecular mechanism of AP in the TNF-α-induced vascular inflammation process in primary cultured human umbilical vein endothelial cells (HUVEC).

## 2. Results and Discussion

**Figure 1:**
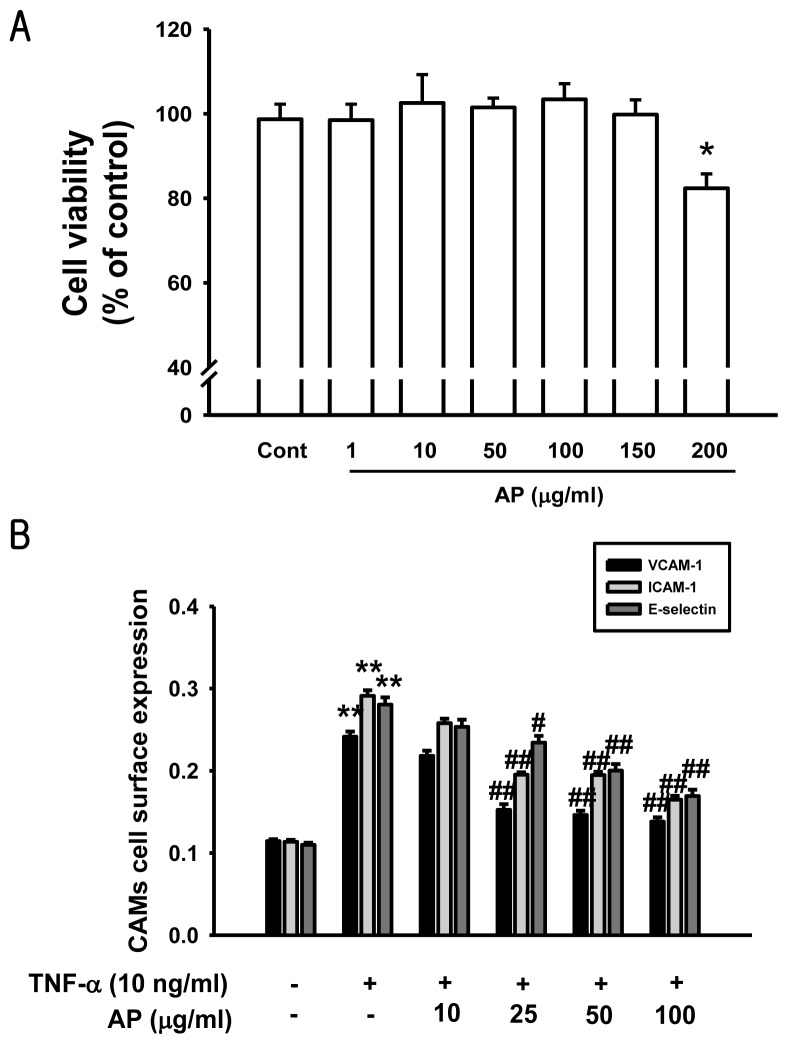
Effects of an aqueous extract of *Portulaca oleracea* (AP) on cytotoxicity and endothelial cell surface expressions of vascular cell adhesion molecules (VCAM-1), intercellular adhesion molecules (ICAM-1), and endothelial cell selectin (E-selectin). (**a**) Confluent endothelial cell monolayers were incubated with various concentrations of AP for 24 h. Cell viability was determined by MTT assay; (**b**) The endothelial cells were pretreated with AP and then stimulated with pro-inflammatory cytokine, tumor necrosis factor (TNF)-α (10 ng/mL) for 6 h. Cell surface expressions of ICAM-1, VCAM-1, and E-selectin were analyzed by cell ELISA, as described in the Materials and Methods. Values are expressed as mean ± S.E. of three individual experiments. * *p* < 0.05, ** *p* < 0.01 *vs.* control, ^##^ *p* < 0.01 *vs.* TNF-α alone.

**Figure 2:**
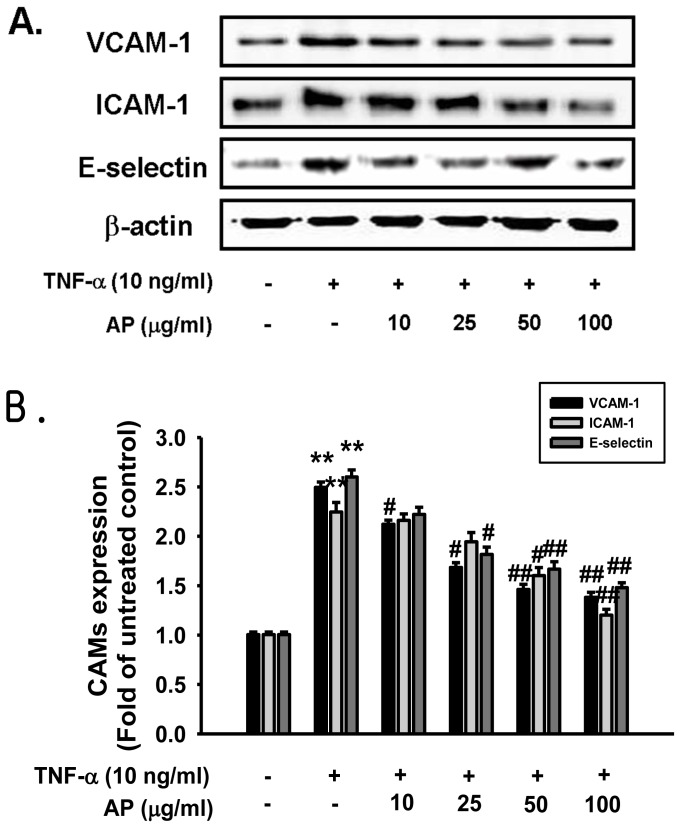
Effect of AP on TNF-α-induced increases in protein expression of VCAM-1, ICAM-1, and E-selectin. (**a**) Primary cultured human umbilical vein endothelial cells (HUVECs) were pretreated with AP and then stimulated with TNF-α for 6 h. After treatment, protein was extracted from the cells. VCAM-1, ICAM-1 and E-selectin protein levels were determined by western blot analysis; (**b**) Quantitative data are expressed as VCAM-1 (black), ICAM-1 (gray), and E-selectin (dark gray) normalized to β-actin, and the results are expressed as the fold of the control. Each electrophoretogram is representative of the results from five independent experiments. * *p* < 0.05, ** *p* < 0.01 *vs.* control, ^#^ *p* < 0.05, ^##^ *p* < 0.01 *vs.* TNF-α alone.

**Figure 3:**
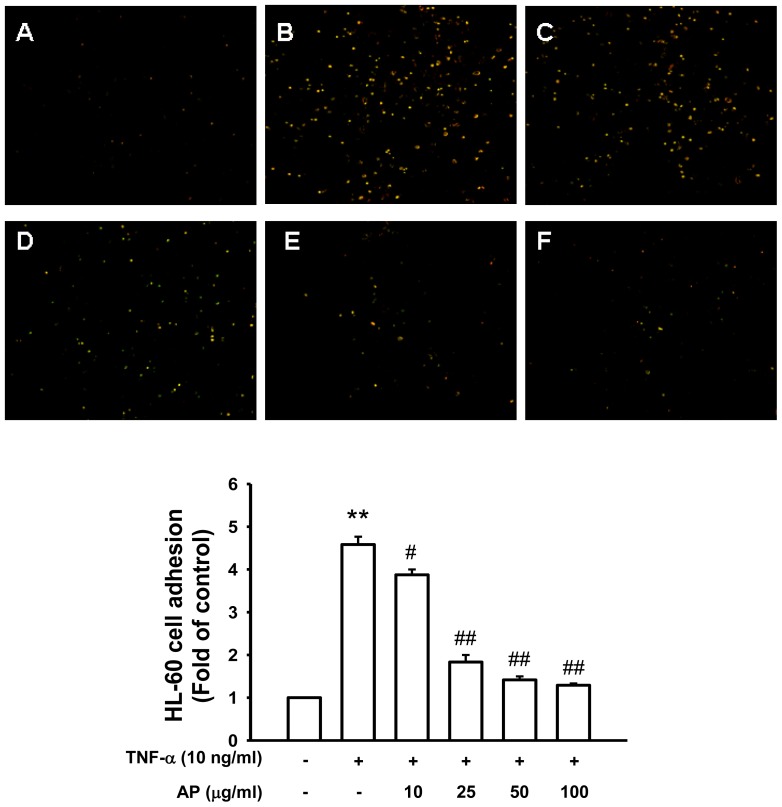
Effect of AP on TNF-α-induced adhesion of HL-60 cells to HUVEC. Adhesion of fluorescence-labeled HL-60 cells to HUVEC was determined as described in Materials and Methods. (**a**) Control; (**b**) TNF-α (10 ng/mL); (**c**) co-treated with TNF-α and AP (10 μg/mL); (**d**) co-treated with TNF-α and AP (25 μg/mL); (**e**) co-treated with TNF-α and AP (50 μg/mL); (**f**) co-treated with TNF-α and AP (100 μg/mL). The amounts of adherent HL-60 cells were monitored by fluorescence microscopy. Low panel represents ratio of fluorescence intensity. Values are expressed as mean ± S.E. of five independent experiments with triplicate dishes. ** *p* < 0.01 *vs.* control, ^#^ *p* < 0.05, ^##^ *p* < 0.01 *vs.* TNF-α alone.

The major finding of this study indicated that pretreatment with AP significantly suppressed the TNF-α-induced intracellular ROS production and up-regulation of adhesion molecules in HUVECs by inhibiting the NF-κB signaling pathway. The cytotoxicity was examined using an MTT assay, performed at 1–200 μg/mL concentration of AP. When incubated with AP (1–200 μg/mL) for 24 h, cell viability did not show significant difference at each concentration; cytotoxicity observed at 200 μg/mL AP (Figure 1a). Therefore 100 μg/mL AP concentration was used as the maximum dose. Increased expression of cell adhesion molecules is an important aspect of inflammatory changes associated with atherosclerosis and contributes to the activation and recruitment of lymphocyte from adventitial vessels and the arterial lumen to the vessel wall [2]. Various agonists including cytokines such as TNF-α and chemokines induce endothelial cell activation. TNF-α stimulation of endothelial cells activates the cell surface expression of adhesion molecules such as ICAM-1, VCAM-1 and E-selectin [6,23]. The effects of AP on the VCAM-1, ICAM-1 and E-selectin expressions in TNF-α-induced HUVEC were determined by ELISA. This data indicated that ICAM-1, VCAM-1, and E-selectin were expressed at low levels on unstimulated endothelial cells. However, treatment with cells to TNF-α (10 ng/mL) for 6 h led to a marked increase of the surface expression of these cell adhesion molecules. Pretreatment with AP significantly inhibited the TNF-α-induced cell surface expressions of VCAM-1 and ICAM-1 in a dose-dependent manner. However, E-selectin expression did not show significant difference (Figure 1b). The inhibitory effect of AP on the expression of the cell adhesion molecules was further confirmed by western blot analysis. As shown in Figure 2, the unstimulated HUVEC expressed low levels of ICAM-1 and VCAM-1 and E-selectin. Upon stimulation with TNF-α, a substantial increase in the expression of all these three molecules was observed. Pretreatment with AP significantly inhibited the TNF-α-induced expression of ICAM-1, VCAM-1 and E-selectin in a dose-dependent manner (Figure 2a,b). Thus, AP inhibited the TNF-α-induced expression of cell adhesion molecules as measured using ELISA and confirmed by western blot analysis. Furthermore, the adhesion of leukocytes or monocytes to the vascular endothelium is an important step in the reaction to inflammation and development of atherosclerotic lesions. During early stages of atherosclerosis, circulating monocytes adhere to endothelial cells that line the vessel wall and transmigrate through the endothelium into the intimal extracellular matrix [24]. The recent study demonstrated that a small number of monocytes adhered to unstimulated HUVECs [25], whereas there was a marked increase in monocyte adherence to HUVECs that had been exposed to TNF-α [26]. To explore the effect of AP on endothelial cell-leukocyte interaction, we examined the adhesion of HL-60 cells to TNF-α-activated HUVEC under static conditions. HUVEC were pretreated with different concentrations of AP (10–100 μg/mL) and the intensity of cell adhesion was evaluated by the quantification of BCECF-AM staining method. Corresponding with results from ELISA, the adhesion of HL-60 cells to TNF-α-stimulated HUVECs was increased about 4.5-fold compared with the untreated cells, and this adhesion was markedly decreased by treatment with AP in a dose-dependent manner (Figure 3). Therefore, we found that treatment with AP significantly reduced monocyte adhesion to TNF-α-induced HUVEC, which is due to the inhibition of adhesion molecules expression. These results suggested that AP could suppress the early pathogenesis of atherosclerosis by regulating the vascular inflammatory process.

**Figure 4:**
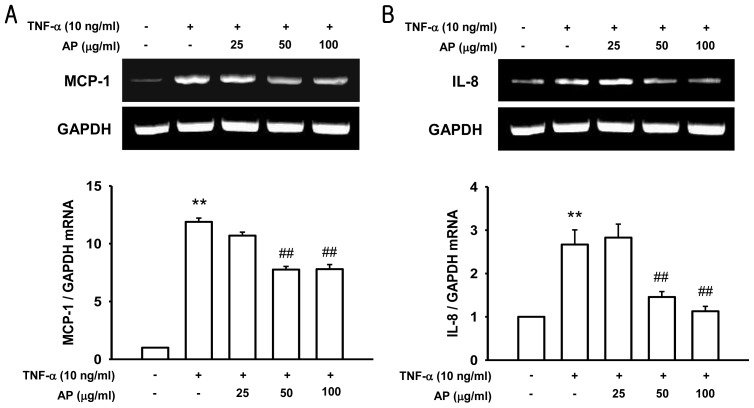
Effect of AP on TNF-α-induced increase of monocyte chemoattractant protein-1 (MCP-1) (**a**) and Interleukin-8 (IL-8) (**b**) mRNA expression. HUVEC were pretreated with AP and then stimulated with TNF-α. MCP-1 and IL-8 mRNA expression was determined by RT-PCR. Values are expressed as a fold of basal value and are the mean ± S.E. of five independent experiments. ** *p* < 0.01 *vs.* control, ^##^ *p* < 0.01 *vs.* TNF-α alone.

Induction of chemotactic cytokines such as IL-8 and MCP-1 is thought to play a key role in monocyte recruitment and adhesion to endothelial cells in atherosclerosis [27]. Previous studies have shown that chemokines and adhesion molecules are involved in causing atherosclerosis by promoting directed migration of inflammatory cells. Interleukin-8 (IL-8) is a C–X–C chemokine and acts as a chemoattractant of neutrophils, whereas monocyte chemoattractant protein-1 (MCP-1) is a C–C chemokine and functions mainly as a chemoattractant of monocytes/macrophages [28]. Both factors are secreted from endothelial cells and have been implicated in the processes leading to atherosclerosis. To understand the mechanisms responsible for inhibition of MCP-1 and IL-8 expression by AP, we examined whether AP blocks the induction of their transcript levels. We performed a RT-PCR analysis on isolated RNA in HUVEC. This data indicated that the unstimulated endothelial cells had low levels of MCP-1 and IL-8 mRNA expression. Stimulation with TNF-α induced a marked increase in MCP-1 and IL-8 mRNA levels, while pretreatment with AP led to a significant reduction in their transcript levels (Figure 4). These results indicated that AP inhibited the transcription of MCP-1 and IL-8 genes under cytokine stimulation. Therefore, AP may be effective in suppression of the vascular inflammatory process associated with the development of atherosclerosis.

**Figure 5:**
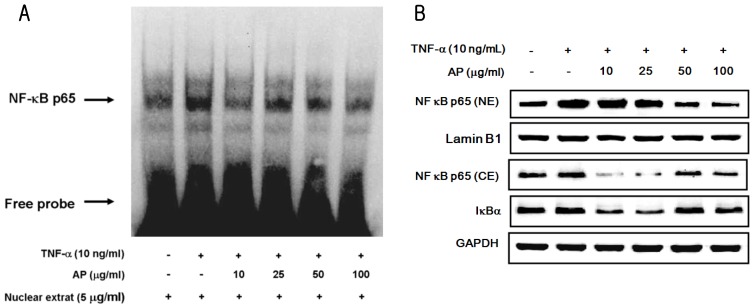
Effect of AP on TNF-α-induced NF-κB activation in HUVECs. Cells were preincubated with AP and treated with 10 ng/mL of TNF-α. (**a**) Nuclear protein extracts were prepared, and electrophoretic mobility shift assay (EMSA) was performed using the biotin-labeled double-stranded oligonucleotide containing consensus NF-κB binding sequences, as described in Materials and Methods; (**b**) Effect of AP on TNF-α-induced NF-κB p65 translocation into nucleus and IκBα degradation in HUVEC. Each electrophoretogram is representative of the results from three independent experiments.

**Figure 6:**
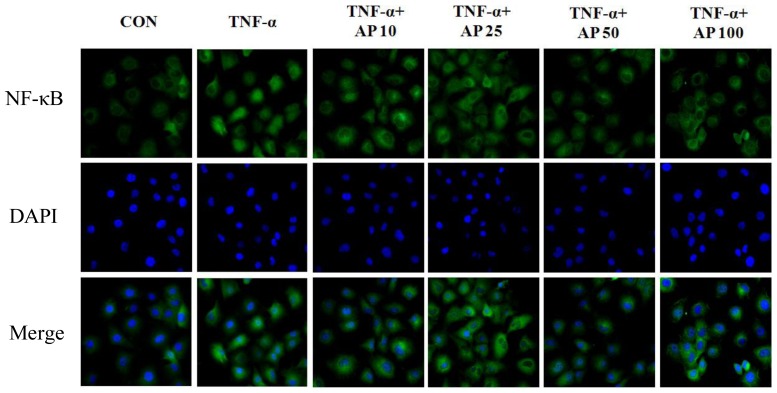
Effect of AP on TNF-α-induced NF-κB nuclear translocation. Subcellular localization of the p65 NF-κB subunit was assayed with the cell immunofluorescence technique. HUVEC were stained with an antibody versus NF-κB (FITC; green staining), and nuclei were counterstained with DAPI (blue staining). Each electrophoretogram is representative of the results from three individual experiments.

It has been also shown that activation of the transcription factor NF-κB by TNF-α is required for the transcriptional activation of endothelial cell adhesion molecules [29]. Nuclear factor κB (NF-κB) has been implicated in the transcriptional activation of numerous genes, including those relevant to atherogenesis and inflammatory responses [30,31]. NF-κB is found in an inactive form in the cytoplasm and appears associated with its inhibitor molecule (I-κB). On stimulation, rapid phosphorylation and degradation of IκBα allows NF-κB to translocate into the nucleus and regulate transcription of the target genes. In cultured human and bovine vascular endothelial cells, p50/p65 heterodimers of NF-κB appear to play a major role in cytokine-dependent transcription of E-selectin, ICAM-1, and VCAM-1 genes [32]. Furthermore, activation of NF-κB occurs in the vascular endothelium covering atherosclerotic lesions in humans [33]. To study the effect of AP on NF-κB activation in TNF-α-induced HUVEC, electrophoretic mobility shift assay (EMSA) was performed. As shown in Figure 5a, stimulation with TNF-α increased the level of NF-κB-DNA-binding activity. This finding was caused by significant retardation in the mobility of the labeled oligonucleotide. In contrast, AP markedly inhibited the NF-κB-DNA-binding activity in the nuclear extract from TNF-α-induced HUVECs. These results therefore, showed that AP inhibits the TNF-α-induced NF-κB activation. Using western blot analysis, NF-κB p65 protein was expressed abundantly in cytoplasm but less in the nucleus of unstimulated cells. However, translocation of NF-κB p65 from the cytoplasm to the nucleus was significantly increased in TNF-α-stimulated cells. TNF-α-dependent translocation of NF-κB p65 to the nucleus was markedly suppressed by pretreatment with AP. In contrast, NF-κB p65 protein in the cytosolic extract significantly increased following treatment with AP. Thus, AP inhibited the TNF-α-induced increase of NF-κB p65 expression levels. Pretreatment with AP inhibited IκBα degradation in a dose dependent manner. Upon induction with TNF-α, the intensity of IκBα was significantly reduced. In contrast, pretreatment of cells with AP significantly inhibited the degradation of IκBα in TNF-α-induced HUVECs (Figure 5b). This was further confirmed by fluorescence microscopy showing that while cells are in a quiescent state, NF-κB p65 protein was mainly located in cytoplasm and only a few cells showed a strongly positive nuclear stain, whereas TNF-α profoundly increased the number of nuclei for the p65 NF-κB. However, AP-treated cells represented the diminished staining level of nuclear p65 in a dose dependent manner (Figure 6).

**Figure 7:**
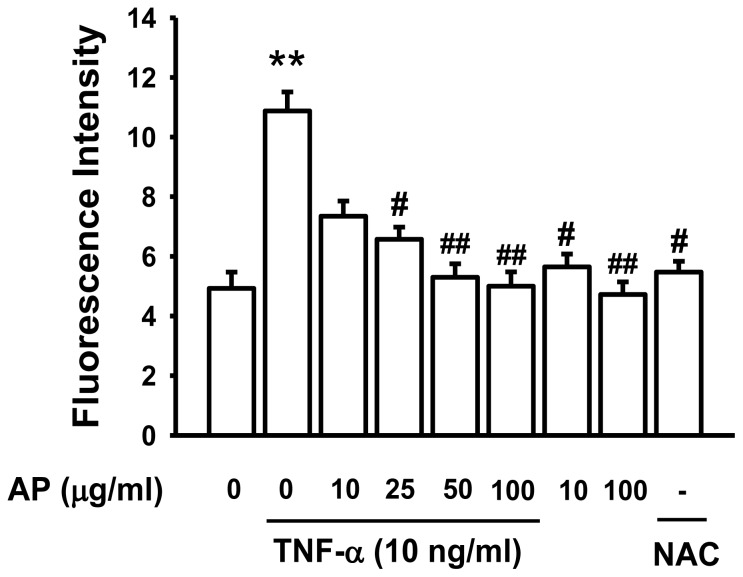
Effect of AP on TNF-α-induced ROS production. HUVECs were pretreated with AP (10–100 μg/mL) or NAC (50 μM) for 30 min and then stimulated with/without TNF-α (10 ng/mL) for 1 h. The fluorescence intensity of cells was measured using a fluorescence microplate. Values are expressed as a mean percentage of the fluorescence intensity ± S.E. of five individual experiments. ** *p* < 0.01 *vs.* control, ^#^ *p* < 0.05, ^##^ *p* < 0.01 *vs.* TNF-α alone.

Emerging evidence suggests that TNF-α signaling cascade may cause oxidative stress due to the production of reactive oxygen species, which in turn temporally regulate NF-κB activity by IκB kinase with subsequent degradation of IκBα [34,35]. Also, a number of antioxidants and free radical quenchers have also been shown to block the NF-κB activation. Reactive oxygen species (ROS) may up-regulate pro-inflammatory gene expression by activating NF-κB, a process that is itself sensitive to the cellular redox state [36]. Therefore, there has been increasing interest in developing the potential therapeutic value of ROS-antioxidants as inhibitors of NF-κB from various plants [37]. To examine the protective effect of AP on TNF-α-induced intracellular ROS production in HUVECs, we measured DCF-sensitive cellular ROS. This data showed that the intracellular ROS production was significantly increased by stimulation of TNF-α. Pretreatment with AP dose-dependently suppressed the TNF-α-induced ROS production in HUVECs (Figure 7). These result suggested that antioxidative activity of AP could contribute in part to its inhibitory effects. Several studies have reported that the oxidative stress might contribute to NF-κB-dependent inflammatory responses. Recent studies reported that antioxidant NAC suppresses vascular NF-κB activation, and this inhibition reduces the pathological thickening of the arterial wall [38]. NF-κB has been shown to be ROS-sensitive. Therefore, we suggest that the antioxidant property of AP was able to inhibit the TNF-α-induced activation of NF-κB.

Recently, our published data suggested that *P. oleracea* ameliorates both vascular inflammation and diabetic nephropathy in db/db mice [39]. The major bioactive components of *Portulaca oleracea* are flavonoids, coumarins, monoterpene glycoside and alkaloids. Furthermore, *P. oleracea* is a rich source of omega-3 fatty acids, gallotannins, kaempferol, quercetin, apigenin, and glutathione [40,41]. However, the mechanism of action and its compounds have not been clarified [42]. Recently, it has been reported that betacyanins from *Portulaca oleracea*, anthocyanins, ameliorate cognition deficits and attenuate oxidative damage in the brains of senescent mice [43]. Generally, polysaccharides and flavones are the usual anti-diabetic compounds in herbs [44,45]. We speculate that various polysaccharides or flavones could participate in the inhibitory effect of vascular inflammation. Thus, further work on isolation and purification of each compound from AP will be done to identify the hypoglycemic effective compounds.

## 3. Experimental Section

### 3.1. Extraction of *Portulaca oleracea*

The *Portulaca oleracea* was purchased from the Herbal Medicine Co-operative Association in Jeonbuk Province, Korea. Herbarium voucher specimen (No. HBE121) was deposited in the herbarium of the Professional Graduate School of Oriental Medicine, Wonkwang University, Iksan, Jeonbuk, South Korea. Dried aerial parts of *P. oleracea* (400 g) were extracted with 5.5 L of boiled distilled water at 100 °C for 2 h. The aqueous extract was centrifuged at 1000 *g* for 20 min at 4 °C and the supernatant was filtered with a Whatman No.3 filter paper, and then concentrated using a rotary evaporator. The supernatant extract was lyophilized to produce a powder, which was then kept at 4 °C until use in the experiments. The yield of the water extract of *P. oleracea* was approximately 22.8% of plant powder.

### 3.2. Cell Culture

Primary cultured HUVEC and endothelial cell growth medium (EGM-2) containing 2.5% fetal bovine serum (FBS) and growth supplements were purchased from Cambrex (East Rutherford, NJ). HUVEC which were used between passages three and eight were maintained in EGM-2 in a humidified chamber containing 5% CO_2_ at 37 °C.

### 3.3. Determination of Cell Based ELISA

ELISA was used to determine the level of ICAM-1, VCAM-1, and E-selectin expression on the cell surface, as previously described with minor modifications [46]. Briefly, HUVEC were fixed by 1% paraformaldehyde and exposed to mouse anti-human ICAM-1, VCAM-1, or E-selectin antibodies at 1:1000 dilution in phosphate-buffered saline (PBS) containing 1% bovine serum albumin (BSA) for 2 h at room temperature. The cells were washed and incubated with a horseradish peroxidase (HRP)-conjugated secondary antibody. The expression of VCAM-1, ICAM-1, or E-selectin was quantified by adding a peroxidase substrate solution (40 mg *o*-phenylenediamine and 10 μL 30% H_2_O_2_ in 100 mL 0.05 M citrate-phosphate buffer). After incubation for 30 min at 37 °C, the reaction was stopped by addition of 5 N H_2_SO_4_, and the absorbance of each well was measured at 490 nm by a Multiskan microplate reader (Thermo LabSystems Inc., Franklin, MA).

### 3.4. Monocyte-Endothelial Cell Adhesion Assay

The cell adhesion assay was modified as described [47]. Briefly, regularly passaged HL-60 cells were labeled with 10 μg/mL 2′,7′-bis-(carboxyethyl)-5,6-carboxyfluorescein acetoxymethyl ester (BCECF/AM, Sigma Chemical Co., St. Louis, MO) at 10 μM final concentration in RPMI-1640 medium containing 10% FBS at 37 °C for 30 min. The labeled cells were harvested by centrifugation and washed three times with PBS before suspension in the medium, and added to HUVEC in six-well culture plates at 4 × 10^5^ cells/mL. The co-incubation was done at 37 °C for 1 h and nonadhering HL-60 cells (American Type Culture Collection, Manassas, VA) were removed by stringent washing two times with PBS. HL-60 cells bound to HUVEC were measured by fluorescence microscopy (Leica DMIRB, Leica, Germany) and were lysed with 50 mM Tris-HCl, pH 8.0, containing 0.1% sodium dodecyl sulfate (SDS). The fluorescent intensity was measured using a spectrofluorometer (F-2500, Hitachi, Tokyo, Japan) at an excitation and emission wavelength of 485 nm and 535 nm, respectively. The adhesion data are represented in terms of the percentage change compared with the control values.

### 3.5. Preparation of Cytoplasmic and Nuclear Extracts

The cells were rapidly harvested by sedimentation and nuclear and cytoplasmic extracts were prepared on ice as previously described by the method of Mackman *et al.* [19]. Cells were harvested and washed with 1 mL buffer A (10 mM HEPES, pH 7.9, 1.5 mM MgCl_2_, 19 mM KCl) for 5 min at 600 *g*. The cells were then resuspended in buffer A and 0.1% NP 40, left for 10 min on ice to lyse the cells and then centrifuged at 600 *g* for 3 min. The supernatant was saved as cytosolic extract. The nuclear pellet was then washed in 1 mL buffer A at 4200 *g* for 3 min, resuspended in 30 μL buffer C (20 mM HEPES, pH 7.9, 25% glycerol, 0.42 M NaCl, 1.5 mM MgCl_2_, 0.2 mM EDTA), rotated for 30 min at 4 °C, then centrifuged at 14,300 *g* for 20 min. The supernatant was used as nucleus extract.

### 3.6. Protein Extraction and Western Blot Analysis

Cell homogenates (40 μg of protein) were separated on 10% SDS-polyacrylamide gel electrophoresis and transferred to nitrocellulose paper. Blots were then washed with H_2_O, blocked with 5% skimmed milk powder in Tris-Buffered Saline Tween-20 (TBST) (10 mM Tris-HCl, pH 7.6, 150 mM NaCl, 0.05% Tween-20) for 1 h, and incubated with the appropriate primary antibody at dilutions recommended by the supplier. Then the membrane was washed and primary antibodies were detected with goat anti-rabbit-IgG or rabbit anti-mouse-IgG conjugated to horseradish peroxidase, and the bands were visualized with enhanced chemiluminescence (Amersham Bioscience, Buckinghamshire, UK). Protein expression levels were determined by analyzing the signals captured on the nitrocellulose membranes using the ChemiDoc image analyzer (Bio-Rad Laboratories, Hercules, CA).

### 3.7. Electrophoretic Mobility Shift Assay

Electrophoretic mobility shift assay (EMSA) for NF-κB was performed using TM a lightshiftchemiluminescent EMSA kit (Pierce, Rockford, IL, USA) by following the manufacturer’s protocol. To start with, DNA was biotin labeled using the Biotin 3′ endlabeling kit (Pierce, Rockford, IL, USA). Briefly, in a 50 μL reaction buffer, 5 pmol of double-stranded NF-κB oligonucleotide 5′-GAT CTC AGA GGG GAC TTT CGA GAG A-3′; 3′-CTA GAG TCT CCC CTG AAA GGC TCT CT-5′ was incubated in a microcentrifuge tube with 10 μL of 5·terminal deoxynucleotidyl transferase (TdT) buffer, 5 μL of 5 M biotin-N4-CTP, 10 U of diluted TdT, and 25 μL of ultrapure water and incubated at 37 °C for 30 min. The reaction was stopped with 2.5 μL of 0.2 M EDTA. To extract labeled DNA, 50 μL of chloroform: isoamyl alcohol (24:1) was added to each tube and centrifuged briefly at 13,000 rpm. The top aqueous phase containing the labeled DNA was removed and saved for binding reactions. Each binding reaction contained 1× binding buffer (100 mM Tris, 500 mM KCl, 10 mM dithiothreitol, pH 7.5), 2.5% glycerol, 5 mM MgCl_2_, 50 ng/μL poly (dI-dC), 0.05% NP-40, 5 μg of nuclear extract and 20–50 fm of biotin-endlabeled target DNA. The content was incubated at room temperature for 20 min. To this reaction mixture, 5 μL of 5× loading buffer was added, subjected to gel electrophoresis on a native polyacrylamide gel, and transferred to a nylon membrane. When the transfer was complete, DNA was crosslinked to the membrane at 120 mJ/cm^2^ using a UV crosslinker equipped with 254 nm bulbs. The biotin-endlabeled DNA was detected using streptavidin-HRP conjugate and a chemiluminescent substrate. The membrane was exposed to using digitalized scientific software program Quantity One^®^ (Silk Scientific Corporation, Orem, UT, USA).

### 3.8. RNA Isolation and RT-PCR Analysis

Total RNA was isolated from cultured endothelial cells using a commercially available kit (RNeasy Mini Kit; Qiagen, Valencia, CA, USA). Briefly, cells were washed with PBS, and then RLT buffer (containing 10% β-mercaptoethanol) was used. Supernatants were transferred into RNase-free microcentrifuge tubes, 70% ethanol was added, and the mixture was applied to a Total RNA Mini column, centrifuged, and the flow-through discarded. Bound RNA in the column was eluted by RNase-free ddH_2_O, and RNA was stored at −80 °C. The OD_260_ and OD_260/280_ values were measured with a spectrophotometer to determine the RNA concentrations. Briefly, in the first step, cDNA was prepared from 500 ng RNA by reverse transcription in a final volume of 20 μL in an Opticon MJ Research instrument. The samples were incubated at 37 °C for 60 min and 94 °C for 5 min. cDNA was stored at −20 °C. cDNA samples were analyzed for the specific cDNA of VCAM-1, MCP-1, IL-8, and GAPDH by PCR amplification using specific primers (Table 1). Template cDNA and 50 nM primers were placed in PCR Pre-mix according to the manufacturer’s specification (Bioneer, Korea). The amplification profile was as follows: an initial cycling at 94 °C for 15 min followed by 45 cycles of 94 °C, 20 s; 60 °C, 20 s; 72 °C, 30 s and a final extension of 72 °C for 5 min. The PCR products were subjected to 1.2% agarose gel electrophoresis. Quantitative data normalized to GAPDH were obtained from a densitometer and analyzed with the included Quantity One software (Silk Scientific Corporation, Orem, UT, USA).

### 3.9. Immunofluorescence Microscopy

HUVECs were seeded on sterile slide coverslips in 6 well plates overnight and pretreated with AP before being stimulated with TNF-α. After several washes with PBS, cells were fixed with 4% paraformaldehyde for 30 min at room temperature and permeabilized with 0.1% Triton-100 in PBS for 30 min. The cells were overlaid with protease-free BSA for 10 min, rinsed with PBS and incubated with anti-NF-κB p65 (Santa Cruz Biotechnology, CA, USA) for 1 h at room temperature. Cells were washed three times with PBS and then incubated with FITC-conjugated goat anti-rabbit IgG as the secondary antibody (Sigma, St Louis, MO, USA). The slides were incubated at 37 °C for 45 min, and nuclear staining was performed with DAPI (Molecular Probe, Eugene, OR, USA). Cells were finally washed three times with PBS, coverslips were mounted with Dako Fluorescent mounting medium onto glass slides, and examined under a fluorescence microscope (Axiovision 4, Zeiss, Germany).

### 3.10. Intracellular ROS Production Assay

The fluorescent probe, 5-(and-6)-chloromethyl-2′,7′-dichlorodihydro-fluorescein diacetate (CM-H_2_DCF-DA), was used to determine the intracellular generation of ROS by stimulation of TNF-α. Briefly, the confluent HUVECs in the 24-well plates were pretreated with AP for 30 min. After removing the AP from the wells, the cells were incubated with 20 μM DCF-DA for 30 min. The cells were then stimulated with TNF-α (10 ng/mL), and the fluorescence intensity (relative fluorescence units) was measured at an excitation and emission wavelength of 485 nm and 530 nm, respectively, using a fluorescence spectrophotometer (F-2500, Hitachi, Tokyo, Japan).

### 3.11. Statistical Analysis

Values were expressed as mean ± S.E. Statistical analyses were performed using analysis of variance followed by the Student’s *t*-test and one-way ANOVA. Differences with a value of *p* < 0.05 were considered statistically significant.

## 4. Conclusions

Vascular inflammation induced by cytokine occurs early in the development of atherosclerosis, and leads to endothelial dysfunction. The present study indicated that AP significantly suppressed the following events in cultured vascular endothelial cells; TNF-α-induced intracellular ROS formation and the redox-sensitive NF-κB activation via the suppression IκB degradation and phosphorylation, cell adhesion molecules expression, and the adhesion to monocytes. These results demonstrate that AP has anti-vascular inflammatory activity and AP may be useful in the prevention and treatment of vascular inflammatory diseases. Thus, this data has cast a new light on the actions of *Portulaca oleracea* and its potential benefits in preventing atherosclerosis.

## Figures and Tables

**Table 1:** Oligonucleotide primer used for RT-PCR.

Primer	Sequence
VCAM-1	sense: CAAATCCTTGATACTGCTCATC
	anti-sense: TTGACTTCTTGCTCACAGC
MCP-1	sense: CAGCCAGATGCAATCAATGC
	anti-sense: GTGGTCCATGGAATCCTGAA
IL-8	sense: GCATAAAGACATACTCCAAACC
	anti-sense: ACTTCTCCACAACCCTCTG
GAPDH	sense: GCACCGTCAAGGCTGAGAAC
	anti-sense: TGGTGGTGAAGACGCCAGT

## References

[1] Libby P., Sukhova G., Lee R.T., Galis Z.S. (1995). Cytokines regulate vascular functions related to stability of the atherosclerotic plaque. J. Cardiovasc. Pharmacol.

[2] Ross R. (1999). Atherosclerosis: An inflammatory disease. N. Engl. J. Med.

[3] Quagliaro L., Piconi L., Assaloni R., da Ros R., Maier A., Zuodar G., Ceriello A. (2005). Intermittent HG enhances ICAM-1, VCAM-1 and E-selectin expression in human umbilical vein endothelial cells in culture: The distinct role of protein kinase C and mitochondrial superoxide production. Atherosclerosis.

[4] Minami T., Aird W.C. (2005). Endothelial cell gene regulation. Trends Cardiovasc. Med.

[5] Madge L.A., Pober J.S. (2001). TNF signaling in vascular endothelial cells. Exp. Mol. Pathol.

[6] Ahmad M., Zhang Y., Parpharalambus C., Alexander R.W. (2001). Role of isoprenylcysteine carboxyl methyltransferase in tumor necrosis factor-α stimulation of expression of vascular cell adhesion molecule-1 in ECs. Arterioscler. Thromb. Vasc. Biol.

[7] Ramana K.V., Bhatnagar A., Srivastava S.K. (2004). Inhibition of aldose reductase attenuates TNF-α-induced expression of adhesion molecules in endothelial cells. FASEB J.

[8] Lockyer J.M., Colladay J.S., Alprin-Lea W.L., Hammond T., Buda A.J. (1998). Inhibition of nuclear factor κB-mediated adhesion molecule expression in human endothelial cells. Circ. Res.

[9] Lee Y.J., Kang D.G., Kim J.S., Lee H.S. (2008). Lycopus lucidus inhibits high glucose-induced vascular inflammation in human umbilical vein endothelial cells. Vascul. Pharmacol.

[10] Kim H.J., Park K.G., Yoo E.K., Kim Y.H., Kim Y.N., Kim H.S., Kim H.T., Park J.Y., Lee K.U., Jang W.G. (2007). Effects of PGC-1α on TNF-α-induced MCP-1 and VCAM-1 expression and NF-κB activation in human aortic smooth muscle and endothelial cells. Antioxid. Redox. Signal.

[11] Harrison D., Griendling K.K., Landmesser U., Hornig B., Drexler H. (2003). Role of oxidative stress in atherosclerosis. Am. J. Cardiol.

[12] Chen C.C., Chow M.P., Huang W.C., Lin Y.C., Chang Y.J. (2004). Flavonoids inhibit tumor necrosis factor-α-induced up-regulation of intercellular adhesion molecule-1 (ICAM-1) in respiratory epithelial cells through activator protein-1 and nuclear factor-κB: Structure-activity relationships. Mol. Pharmacol.

[13] Yoon J.J., Lee Y.J., Kim J.S., Kang D.G., Lee H.S. (2010). Protective role of betulinic acid on TNF-α-induced cell adhesion molecules in vascular endothelial cells. Biochem. Biophys. Res. Commun.

[14] Mohanapriya S., Senthilkumar P., Sivakumar S., Dineshkumar M., Subbhuraam C.V. (2006). Effects of copper sulfate and copper nitrate in aquatic medium on the restoration potential and accumulation of copper in stem cuttings of the terrestrial medicinal plant, *Portulaca oleracea* Linn. Environ. Monit. Assess.

[15] Rasheed A.N., Afifi F.U., Shaedah M., Taha M.O. (2004). Investigation of the active constituents of *Portulaca oleracea* L. (Portulacaceae) growing in Jordan. Pak. J. Pharm. Sci.

[16] Zhang X.J., Ji Y.B., Qu Z.h.Y., Xia J.C.h., Wang L. (2002). Experimental studies on antibiotic functions of *Portulaca oleracea* L. *in vitro*. Chin. J. Microecol.

[17] Chan K., Islam M.W., Kamil M., Radhakrishnan R., Zakaria M.N.M., Habibullah M., Attas A. (2000). The analgesic and anti-inflammatory effects of *Portulaca oleracea* L. subsp. sativa (Haw.) Celak. J. Ethnopharmacol.

[18] Parry O., Marks J.A., Okwuasaba F. (1993). The skeletal muscle relaxant action of *Portulaca oleracea*: Role of potassium ions. J. Ethnopharmacol.

[19] Rasheed A.N., Afifi F.U., Disi A.M. (2003). Simple evaluation of the wound healing activity of a crude extract of *Portulaca oleracea* L. (growing in Jordan) in Mus musculus JVI-1. J. Ethnopharmacol.

[20] Awad N.E. (1994). Lipid content and antimicrobial activity of phenolic constituents of cultivated *Portulaca oleracea* L. Bull. Fac. Pharm.

[21] Sakai N., Inada K., Okamoto M., Shizuri Y., Fukuyama Y., Portuloside A. (1996). a monoterpene glucoside from *Portulaca oleracea*. Phytochemistry.

[22] Liu L., Howe P., Zhou Y.F., Xu Z.Q., Hocart C., Zhan R. (2000). Fatty acids and beta-carotene in australian purslane (*Portulaca oleracea*) varieties. J. Chromatogr. A.

[23] Che W., Lerner-Marmarosh N., Huang Q., Osawa M., Ohta S., Yoshizumi M. (2002). Insulin-like growth factor-1 enhances inflammatory responses in ECs: Role of Gab1 and MEKK3 in TNF-α-induced c-Jun and NF-κB activation and adhesion molecule expression. Circ. Rec.

[24] Abou-Raya A., Abou-Raya S. (2006). Inflammation: A pivotal link between autoimmune diseases and atherosclerosis. Autoimmun. Rev.

[25] Sneddon A.A., McLeod E., Wahle K.W., Arthur J.R. (2006). Cytokine-induced monocyte adhesion to endothelial cells involves platelet-activating factor: Suppression by conjugated linoleic acid. Biochim. Biophys. Acta.

[26] Lee D.K., Nathan, Grantham R., Trachte A.L., Mannion J.D., Wilson C.L. (2006). Activation of the canonical Wnt/beta-catenin pathway enhances monocyte adhesion to endothelial cells. Biochem. Biophys. Res. Commun.

[27] Gerszten R.E., Garcia-Zepeda E.A., Lim Y.C. (1999). MCP-1 and IL-8 trigger firm adhesion of monocytes to vascular endothelium under flow conditions. Nature.

[28] Yang Y.Y., Hu C.J., Chang S.M., Tai T.Y., Leu S.J. (2004). Aspirin inhibits monocyte chemoattractant protein-1 and interleukin-8 expression in TNF-α stimulated human umbilical vein endothelial cells. Atherosclerosis.

[29] Collins T., Read M.A., Neish A.S., Whitley M.Z., Thanos D., Maniatis T. (1995). Transcriptional regulation of endothelial cell adhesion molecules: NF-κB and cytokine inducible enhancers. FASEB J.

[30] Murase T., Kume N., Hase T., Shibuya Y., Nishizawa Y., Tokimitsu I., Kita T. (1999). Gallates inhibit cytokine-induced nuclear translocation of NF-κB and expression of leukocyte adhesion molecules in vascular endothelial cells. Arterioscler. Thromb. Vasc. Biol.

[31] Kanters E., Pasparakis M., Gijbels M.J., Vergouwe M.N., Partouns-Hendriks I., Fijneman R.J., Clausen B.E., Förster I., Kockx M.M., Rajewsky K. (2003). Inhibition of NF-κB activation in macrophages increases atherosclerosis in LDL receptor-deficient mice. J. Clin. Invest.

[32] Trivedi C.M., Patel R.C., Patel C.V. (2007). Homeobox gene HOXA9 inhibits nuclear factor-κB dependent activation of endothelium. Atherosclerosis.

[33] Hajra L., Evans A.I., Chen M., Hyduk S.J., Collins T., Cybulsky M.I. (2000). The NF-κB signal transduction pathway in aortic endothelial cells is primed for activation in regions predisposed to atherosclerotic lesion formation. Proc. Natl. Acad. Sci. USA.

[34] Kumar S., Sharma A., Madan B., Singhal V., Ghosh B. (2007). Isoliquiritigenin inhibits IκB kinase activity and ROS generation to block TNF-α induced expression of cell adhesion molecules on human endothelial cells. Biochem. Pharmacol.

[35] Bonizzi G., Piette J., Merville M.P., Bours V. (2000). Cell type-specific role for reactive oxygen species in nuclear factor-κB activation by interleukin-1. Biochem. Pharmacol.

[36] Schoonbroodt S., Piette J. (2000). Oxidative stress interference with the nuclear factor-κB activation pathways. Biochem. Pharmacol.

[37] Calixto J.B., Campos M.M., Otuki M.F., Santos A.R. (2004). Antiinflammatory compounds of plant origin. Part II. Modulation of pro-inflammatory cytokines, chemokines and adhesion molecules. Planta Med.

[38] Hayashi K., Takahata H., Kitagawa N. (2001). N-acetylcysteine inhibited nuclear factor-κB expression and the intimal hyperplasia in rat carotid arterial injury. Neurol. Res.

[39] Lee A.S., Lee Y.J., Lee S.M., Yoon J.J., Kim J.S., Kang D.G., Lee H.S. (2012). *Portulaca oleracea* ameliorates diabetic vascular inflammation and endothelial dysfunction in db/db mice. Evid. Based Complement Alternat Med.

[40] Radhakrishnan R., Zakaria M.N., Islam M.W., Chen H.B., Kamil M., Chan K. (2001). Neuropharmacological actions of *Portulaca oleracea* L v. sativa (Hawk). J. Ethnopharmacol.

[41] Cai Y., Luo Q., Sun M., Corke H. (2004). Antioxidant activity and phenolic compounds of 112 traditional Chinese medicinal plants associated with anticancer. Life Sci.

[42] Gao D., Li Q., Fan Y. (2010). Hypoglycemic effects and mechanisms of *Portulaca oleracea* L. in alloxan-induced diabetic rats. J. Med. Plants Res.

[43] Wang C.Q., Yang G.Q. (2010). Betacyanins from *Portulaca oleracea* L. ameliorate cognition deficits and attenuate oxidative damage induced by D-galactose in the brains of senescent mice. Phytomedicine.

[44] Zhao R., Li Q., Xiao B. (2006). Effect of *Lycium barbarum* polysaccharide on the improvement of insulin resistance in NIDDM rats. Yakugaku Zassh.

[45] Singab A.N., El-Beshbishy H.A., Yonekawa M., Nomura T., Fukai T. (2005). Hypoglycemic effect of Egyptian Morus alba root bark extract: Effect on diabetes and lipid peroxidation of streptozotocin-induced diabetic rats. J. Ethnopharmacol.

[46] Manduteanu I., Voinea M., Antohe F., Dragomir E., Capraru M., Radulescu L., Simionescu M. (2003). Effect of enoxaparin on high glucose-induced activation of endothelial cells. Eur. J. Pharmacol.

[47] De Clerck L.S., Bridts C.H., Mertens A.M., Moens M.M., Stevens W.J. (1994). Use of fluorescent dyes in the determination of adherence of human leucocytes to endothelial cells and the effect of fluorochromes on cellular function. J. Immunol. Methods.

